# A mechanistic understanding of the effect of *Staphylococcus aureus* VraS histidine kinase single-point mutation on antibiotic resistance

**DOI:** 10.1128/spectrum.00095-25

**Published:** 2025-04-15

**Authors:** Liaqat Ali, Salima Karki, Gunavanthi D. Boorgula, Amir Mekakda, Brittnee Cagle-White, Shrijan Bhattarai, Robert Beaudoin, Aryanna Blakeney, Sanjay Singh, Shashikant Srivastava, May H. Abdel Aziz

**Affiliations:** 1 Fisch College of Pharmacy, University of Texas at Tyler 12347, Tyler, Texas, USA; 2 Division of Infectious Diseases, Department of Medicine, School of Medicine, University of Texas at Tyler 675071, Tyler, Texas, USA; 3 Department of Cellular and Molecular Biology, University of Texas Health Science Center at Tyler 12341, Tyler, Texas, USA; McGill University, Ste-Anne-de-Bellevue, Quebec, Canada

**Keywords:** *Staphylococcus aureus*, VraS, kinase, mutation, antibiotic resistance

## Abstract

Bacterial genomic mutations in *Staphylococcus aureus* have been detected in isolated resistant clinical strains, yet their mechanistic effect on the development of antimicrobial resistance remains unclear. Resistance-associated regulatory systems acquire adaptive mutations under stress conditions that may lead to a gain-of-function effect and contribute to the resistance phenotype. Here, we investigate the effect of a single-point mutation (T331I) in VraS histidine kinase, part of the VraSR two-component system in *S. aureus*. VraSR senses and responds to environmental stress signals by upregulating gene expression for cell wall synthesis. A combination of enzyme kinetics, microbiological, and transcriptomic analyses revealed the mechanistic effect of the mutation on VraS and *S. aureus*. Michaelis-Menten kinetics show that the VraS mutation caused an increase in the autophosphorylation rate of VraS and enhanced its catalytic efficiency. The introduction of the mutation through recombineering coupled with CRISPR-Cas9 counterselection to the Newman strain wild-type (WT) genome doubled the minimum inhibitory concentration of three cell wall-targeting antibiotics. The mutation caused an enhanced *S. aureus* growth rate at sub-lethal doses of the antibiotics, confirming the causative effect of the mutation on bacterial persistence. Transcriptomic analysis showed a genome-wide alteration in gene expression levels and protein-protein interaction network of the mutant compared to the WT strain after exposure to vancomycin. The results suggest that the *vraS* mutation causes several mechanistic changes at the protein and cellular levels that favor bacterial survival under antibiotic stress and cause the mutation-harboring strains to become the dominant population during infection.

**IMPORTANCE:**

Rising antimicrobial resistance (AMR) is a global health problem. Mutations in the two-component system have been linked to drug resistance in *Staphylococcus aureus*, yet the exact mechanism through which these mutations work is understudied. We investigated the T331I mutation in the *vraS* gene linked to sensing and responding to cell wall stress. The mutation caused changes at the protein level by increasing the catalytic efficiency of VraS kinase activity. Introducing the mutation to the genome of an *S. aureus* strain resulted in changes in phenotypic antibiotic susceptibility, growth kinetics, and genome-wide transcriptomic alterations. By a combination of enzyme kinetics, microbiological, and transcriptomic approaches, we highlight how small genetic changes can significantly impact bacterial physiology and survival under antibiotic stress. Understanding the mechanistic basis of antibiotic resistance is crucial to guide the development of novel therapeutic agents to combat AMR.

## INTRODUCTION

*Staphylococcus aureus* is a major pathogenic bacterium that causes serious skin, soft tissue, and nosocomial infections that could progress to severe illnesses, such as osteomyelitis, endocarditis, sepsis, and bacteremia in humans (1). Methicillin-resistant *S. aureus* (MRSA) is the leading cause of nosocomial infections in hospitals and community settings (2). In 2020, during the COVID-19 pandemic, the rate of MRSA infections in the United States increased by 13% compared to that reported in 2019 (3). The Infectious Diseases Society of America MRSA treatment guidelines recommend either vancomycin or daptomycin as first-line agents (4).

The MRSA phenotype is known to arise as a result of acquired external genetic elements of resistance genes like *mecA* (5). The vancomycin-intermediate *S. aureus* (VISA) is characterized by vancomycin minimum inhibitory concentration (MIC) ranging between 4 and 8 µg/mL. Whole genome sequencing revealed that the VISA phenotype may arise due to mutations within various loci of the *S. aureus* genome. These mutations are mainly adaptive responses driven by environmental stress, including antibiotic exposure (6–8). In addition, mutations in VISA have also been reported in the two-component systems (TCSs) network (9, 10). Despite the randomness of the occurrence of the mutations, the antibiotic stress usually selects for specific mutants that would have a differential survival advantage.

The network of TCSs has several sensory roles in bacteria, including cell wall biosynthesis, such as VraSR, WalKR, and GraSR (11–13). These systems initiate a response mostly after exposure to antibiotics that target the cell wall. For instance, *S. aureus* utilizes VraSR (vancomycin resistance-associated two-component system) to respond to glycopeptides or beta-lactam antibiotics activity (14). The VraS histidine kinase is a transmembrane receptor that phosphorylates the response regulator VraR, which then binds to bacterial DNA and activates the *vraSR* regulon and several other genes, including *pbp2*, *murZ*, *sgtB*, *fmtA*, and *lytR*, whose products collectively overcome the activity of cell wallinhibiting antibiotics (15, 16). Previous studies show that vancomycin exposure can upregulate VraSR expression, resulting in enhanced intracellular survival of *S. aureus* (15, 17, 18). A number of point mutations in the *vraSR* regulon have been reported to be associated with VISA (8, 10, 19–21).

Structurally, VraS belongs to the intramembrane-sensing histidine kinases family that consists of a transmembrane domain, dimerization interface with a conserved histidine residue, and an ATP-binding catalytic domain (22). Among these domains, the ATP-binding domain is implicated in the autophosphorylation of VraS (23). Mutations in this domain were linked to increasing resistance to antibiotics (10, 20). A recent study by Sabat et al. (21) revealed that prolonged daptomycin treatment of infective endocarditis with susceptible *S. aureus* led to the emergence of daptomycin resistance, and whole genome sequencing revealed a VraS T331I mutation in the clinical isolate. To further expand the understanding of *vraS* mutations and the associated mechanism of antibiotic resistance, we performed a series of experiments targeting this T331I mutation located in the ATP-binding domain of VraS using a combination of biochemical, microbiological, and transcriptomic techniques. By unraveling the consequences of the mutation on VraS catalytic functions and *S. aureus* at the cellular level, we attempt to uncover the mechanistic basis for developing antibiotic resistance in this highly pathogenic organism.

## RESULTS

### VraS T331I mutation causes an increased autophosphorylation rate and catalytic efficiency

**Fig 1:**
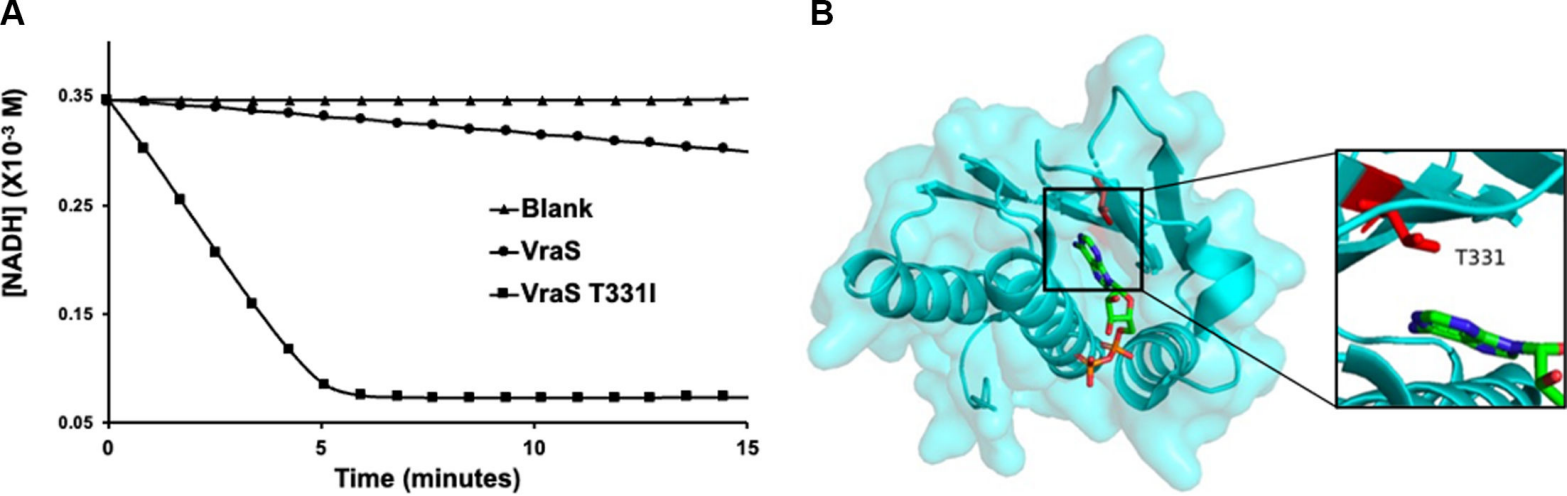
Autophosphorylation of VraS. (**A**) The autophosphorylation reaction rate of VraS WT and T331I mutant (5 µM) as a function of the rate of NADH disappearance, compared to a blank reaction at 2 mM ATP. The reaction reaches a plateau with T331I as the NADH in the assay mixture is depleted. (**B**) Surface representation of the crystallized VraS catalytic domain (PDB 4GT8). The inset shows T331 (red sticks) proximity to the bound ATP (in colored sticks).

The VraS wild-type (WT) construct was amplified from the previously described pGEX-4T-1 plasmid (24). The gene was inserted into a pET15b plasmid and modified to introduce a single-point mutation at the T331 residue as described in the Materials and Methods section. The constructs were expressed in the BL21 (DE3) pLysS strain and purified using Ni-NTA affinity and gel filtration chromatography (Fig. S1). The identity of the proteins was confirmed by Western blotting with anti-His antibody, and a coupled kinase kinetic assay was used to assess the autophosphorylation reaction rate of VraS WT and T331I, as previously described (25). The autophosphorylation rate is a critical aspect of the complex catalytic machinery for histidine kinases and can indicate the efficiency of the system in signal transduction. The reaction rate for T331I was 223.73 ± 5.6 nmol h^−1^ compared to 19.03 ± 1.1 for the WT enzyme, a ~12-fold increase (Fig. 1A). Using increasing ATP concentrations, we performed Michaelis-Menten’s kinetic analysis. The calculated ATP affinity to WT VraS (*K_M_*) was 5.34 ± 1.9 µM compared to 7.95 ± 0.1 µM for the T331I mutant, which was not a significant change. However, the catalytic rate of the reaction (*k*_cat_) increased from 32.02 ± 3.7 to 170.70 ± 2.3 h^−1^, causing the overall catalytic efficiency (*K_M_* /*k*_cat_) of the mutant to increase more than threefold from 6.60 ± 1.7 to 21.37 ± 0.3 µM^−1^ h^−1^ compared to WT VraS. The results can be attributed to the location of the T331 residue in the catalytic binding site in close proximity to the bound ATP (Fig. 1B).

### Shift in minimum inhibitory concentration, drug efficacy, and potency in the VraS T331I mutant

**Fig 2:**
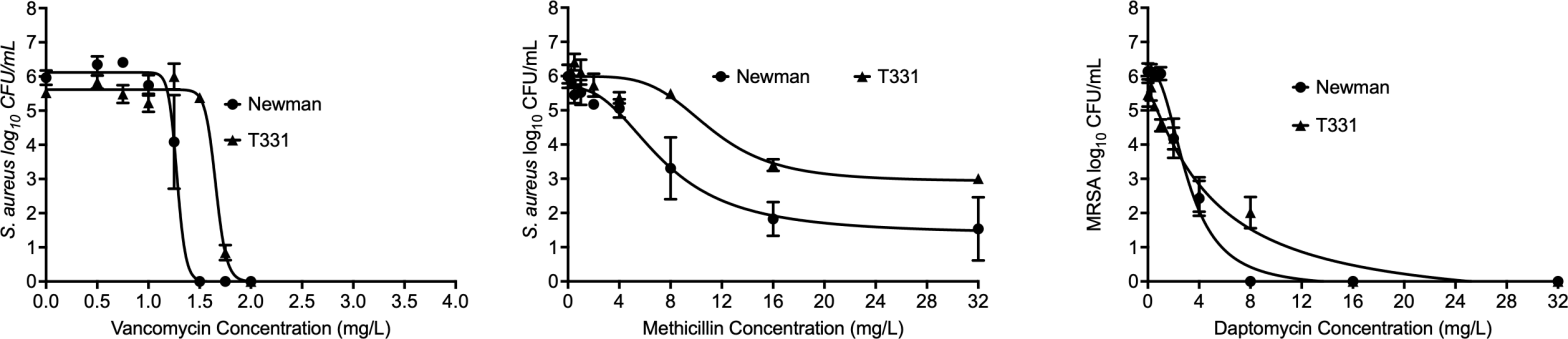
Relationship between the drug concentrations and the bacterial burden. Dose-response curves of the tested antibiotics in the Newman WT and T331I mutant strain. Results obtained are in Table 1, and the parameters for the model are in Table S1.

The T331I mutation was introduced to the genomic background of the *S. aureus-*susceptible Newman WT strain using recombineering techniques with CRISPR-Cas9 counterselection per published protocols (26). Broth microdilution MICs were performed with vancomycin, methicillin, and daptomycin (in the presence of 50 mg/L CaCl_2_) for the WT and the T331I mutant strains in two growth media, cation-adjusted Muller-Hinton broth (CAMHB) and lysogeny broth (LB). The MICs for the T331 mutant show a twofold increase with most of the tested antibiotics (Table 1). Next, concentration-response studies with the three antibiotics were performed using both WT and mutant strains. Data were analyzed using the inhibitory sigmoid maximal effect model to determine the relationship between the drug concentrations and the bacterial burden and are reported here as mean ± standard error (Fig. 2; Table 1; Table S1). Two-way analysis of variance did not show significant differences in the antibiotics’ efficacy (*E*_max_ or maximal effect) and potency (EC_50_ or drug concentration mediating 50% of the *E*_max_). However, the EC_50_ of the mutant strain shows a trend of increasing resistance for all antibiotics compared to the WT strain, indicating the possible role of the *vraS* mutation in antibiotic resistance.

**TABLE 1:** The effect of mutation on drug susceptibility using different media

	MIC (mg/L)				EC_50_ (mg/L) mean ± SE^*a*^	
	CAMHB		LB			
	WT	T331I	WT	T331I	WT	T331I
Vancomycin	1	2	1	2	1.28 ± 0.05	1.66 ± 0.03
Methicillin	8	16	1	2	7.44 ± 1.03	11.14 ± 1.21
Daptomycin	2	2	0.25	0.5	3.10 ± 0.17	5.10 ± 1.03

^*a*^ Standard error.

### The T331I strain demonstrates significant changes in *S. aureus* growth kinetics under antibiotic stress

**Fig 3:**
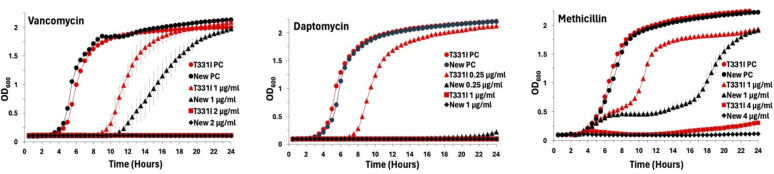
Growth curves of the T331I mutant (red lines) and WT (New) strains (black lines) in the presence of different antibiotic concentrations. PC indicates positive control growth without antibiotics. The data represent the average ± SD of at least three biological replicas.

In the presence of resistant mutant strains of pathogens, the window of concentration between the MIC of the WT to that required to inhibit the least susceptible mutant is expected to enrich the resistant strain subpopulations selectively (27). To assess the extent of this window with the T331I mutation, the growth curves of WT and mutant strains were monitored with and without antibiotic stress, as previously described (24). The strains were grown for 24 h in the presence of different doses of vancomycin, daptomycin, and methicillin. The antibiotic concentrations screened were below to slightly above the MIC for the strains. In the absence of antibiotics, the T331I strain had similar growth kinetics to the WT strain with no significant growth defect. The calculated growth rate for WT Newman was 0.09 (95% CI: 0.08–0.10) log_10_ CFU/mL/h, whereas the T331I was 0.07 (95% CI: 0.06–0.8) log_10_ CFU/mL/h. However, in the presence of antibiotics, the mutant shows faster growth rates, shorter lag time, and more persistence than the WT strain (Fig. 3).

### The expression of *blaZ* is significantly increased in the T331I mutant strain under antibiotic stress

**Fig 4:**
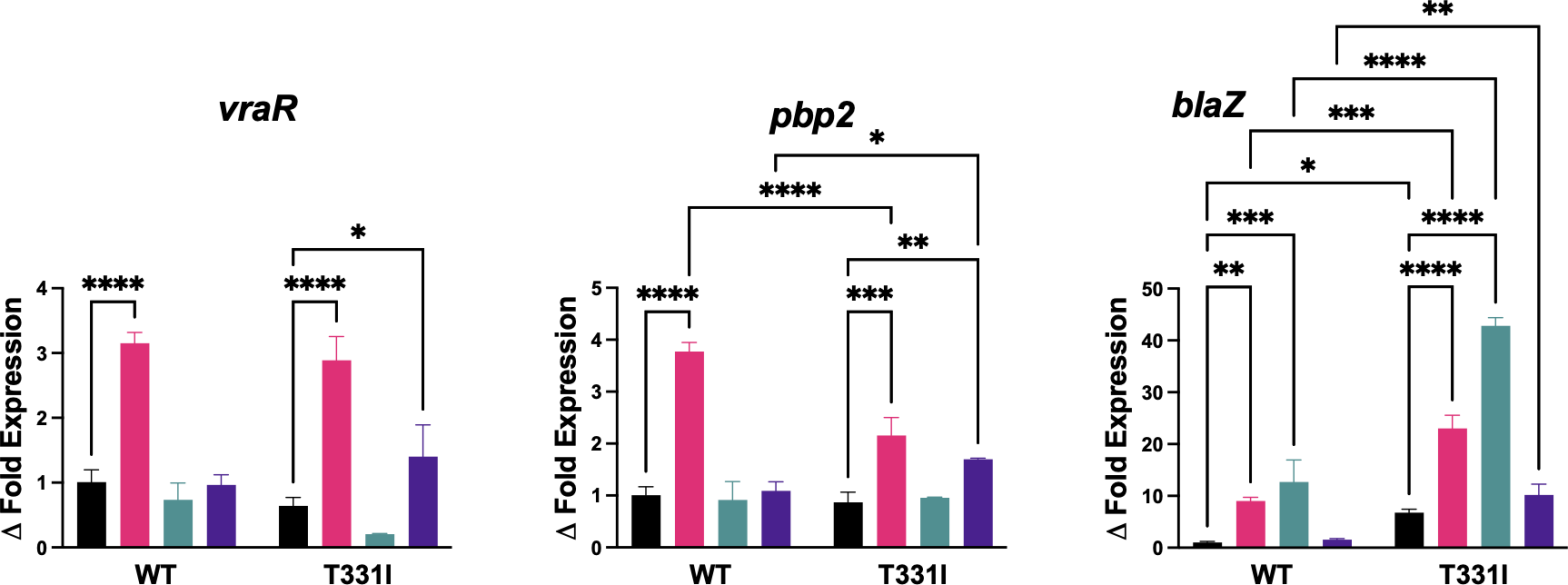
Fold change in the expression levels of *vraR*, *pbp2,* and *blaZ* after mutation and/or antibiotic exposure (black: no antibiotic control, magenta: vancomycin, green: methicillin, and purple: daptomycin). The data represent the mean ± SE (*n*  =  3), and statistical significance between control versus treated samples for each strain and between WT and T331I under the same treatment was calculated via one-way ANOVA with multiple comparisons.

To investigate the possible effect of mutation on VraSR self-regulation and its downstream transcription, we compared the expression of *vraR*, *pbp2*, and *blaZ* in the WT and T331I strains before and after antibiotic stress using quantitative real-time PCR analysis (qRT-PCR), as previously described (24) and detailed in the Materials and Methods section. The *pbp2* gene translates to a transpeptidase that is essential for cell wall formation and highly involved in resistance to antibiotics, while *blaZ* translates to the β-lactamase enzyme responsible for the hydrolysis of β-lactam antibiotics (28). Figure 4 shows the expression of all the tracked genes relative to the Newman WT in the absence of antibiotics. In the absence of antibiotic stress, T331I had similar expression levels to the WT strain for *vraR* and *pbp2* yet showed a 6.5-fold increase in *blaZ* expression (*P* = 0.0209). After vancomycin exposure, the two strains showed a similar trend of increased expression for the tested genes. Compared to the WT, T331I showed a 1.7-fold lower *pbp2* expression (*P* < 0.0001) but a 2.5-fold higher *blaZ* expression (*P* = 0.0001). Methicillin caused an increase only in *blaZ* expression, with T331I showing 3.5-fold higher expression than WT (*P* < 0.0001). Interestingly, daptomycin did not affect the expression level of the tested genes in the WT with all antibiotics. In comparison, T331I had significantly higher expression of *pbp2* (1.5-fold, *P* = 0.0224) and *blaZ* (6.5-fold, *P* = 0.0027) compared to the WT strain.

### Significant transcriptomic changes of the T331I mutant in response to vancomycin exposure

**Fig 5:**
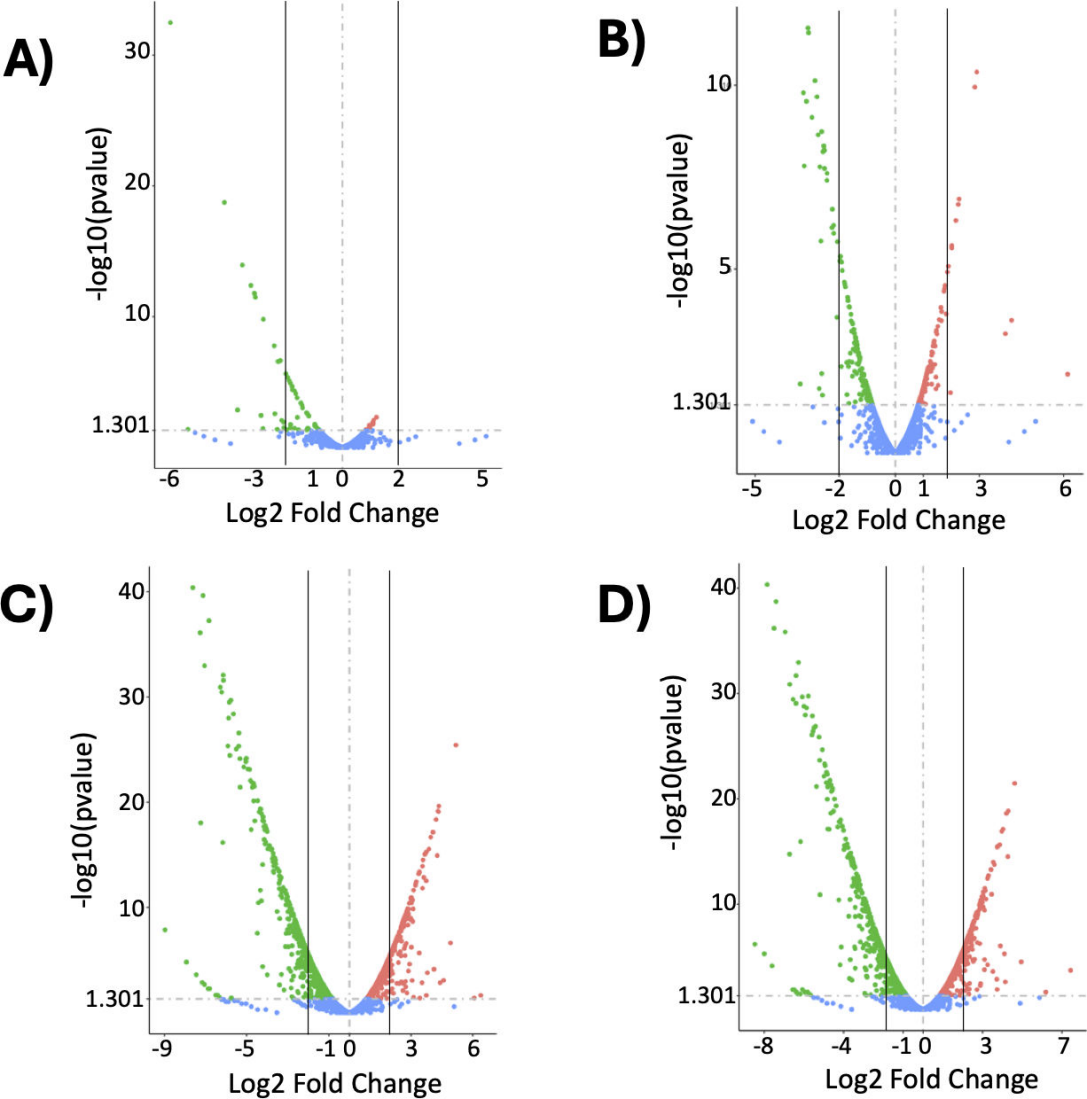
Volcano plots of differential gene expression between T331I and WT strains with and without vancomycin stress. (**A**) T331I compared to WT, (**B**) WT with vancomycin compared to WT, (**C**) T331I with vancomycin compared to T331I, and (D) T331I with vancomycin compared to WT with vancomycin. Colored dots (red, upregulated; green, downregulated; and blue, not significantly changed) based on *P*-value (<0.05) and log2(fold change) and logFC cutoff of 1.3. The vertical lines represent the twofold change in gene expression selected to generate the data in Table 2.

We performed RNA-seq to determine the transcriptomic changes in the T331I mutant compared to the Newman WT before and after vancomycin exposure (2× MIC for 1 h). In the absence of stress, T331I had 70 differentially expressed genes (DEGs), with 22 genes significantly dysregulated by more than twofold compared to the WT strain, representing 0.7% of the detected *S. aureus* genome (Table 2; Fig. 5A). The WT strain showed 287 DEGs in response to vancomycin exposure, including 37 dysregulated genes by more than a twofold change (Table 2; Fig. 5B). Subjecting the T331I mutant strain to the same antibiotic stress resulted in large-scale changes in transcription with >1,200 DEGs compared to T331I in the absence of stress or WT in the presence of the same stress, where in each case >450 genes (~14% of the genome) were dysregulated by more than a twofold change and ~3% by more than a fourfold change (Table 2; Fig. 5C and D). This represents more than a 10-fold change in genome-wide gene expression in response to vancomycin for the mutant strain compared to the WT (the lists of genes can be found in Data S1). The gene expression profile shows that several genes were uniquely expressed in each strain, with and without vancomycin stress (Fig. S2; Data S2).

**TABLE 2:** Comparison of differentially expressed genes in WT and T33I mutant strains (genes are listed in Data S1)

Sample	Reference	No. of DEGs	No. by fold change				% by fold change	
			Upregulated		Downregulated		>|2|	>|4|
			>2	>4	>–2	>–4		
T331I	WT	70	0	0	19	03	0.7	0.1
WT-Van	WT	287	09	02	26	0	1.3	0.1
T331I-Van	T331I	1,246	161	10	211	74	13.7	3.1
T331I-Van	WT-Van	1,356	166	14	218	76	14.1	3.3

To verify the RNA-seq results, qRT-PCR was performed on the top four dysregulated genes to confirm the changes in expression quantitatively using the primers listed in Table S2. The RNA-seq results show that *treP* and *isaA* genes were 5.2- and 4.4-fold upregulated, respectively, in the T331I mutant versus WT under vancomycin stress, while the *hisD* and *cwrA* genes were 7.3- and 6.8-fold downregulated. The dysregulated gene expression trends observed in qRT-PCR were similar to those captured in RNA-seq (Fig. S3). To determine the biological functions of the DEGs in T331I versus WT after vancomycin exposure, a Kyoto Encyclopedia of Genes and Genomes pathway enrichment analysis was performed (29). Pathways involved in the ribosome and amino acid degradation were significantly upregulated. The downregulated pathways include the biosynthesis of amino acids, biosynthesis of secondary metabolites, and 2-oxocarboxylic acid metabolism. The pathway of microbial metabolism in diverse environments was highly dysregulated, with 41 upregulated and 48 downregulated genes (Fig. S4).

### The T331I mutation causes significant changes in the *S. aureus* predicted protein–protein interaction network

**Fig 6:**
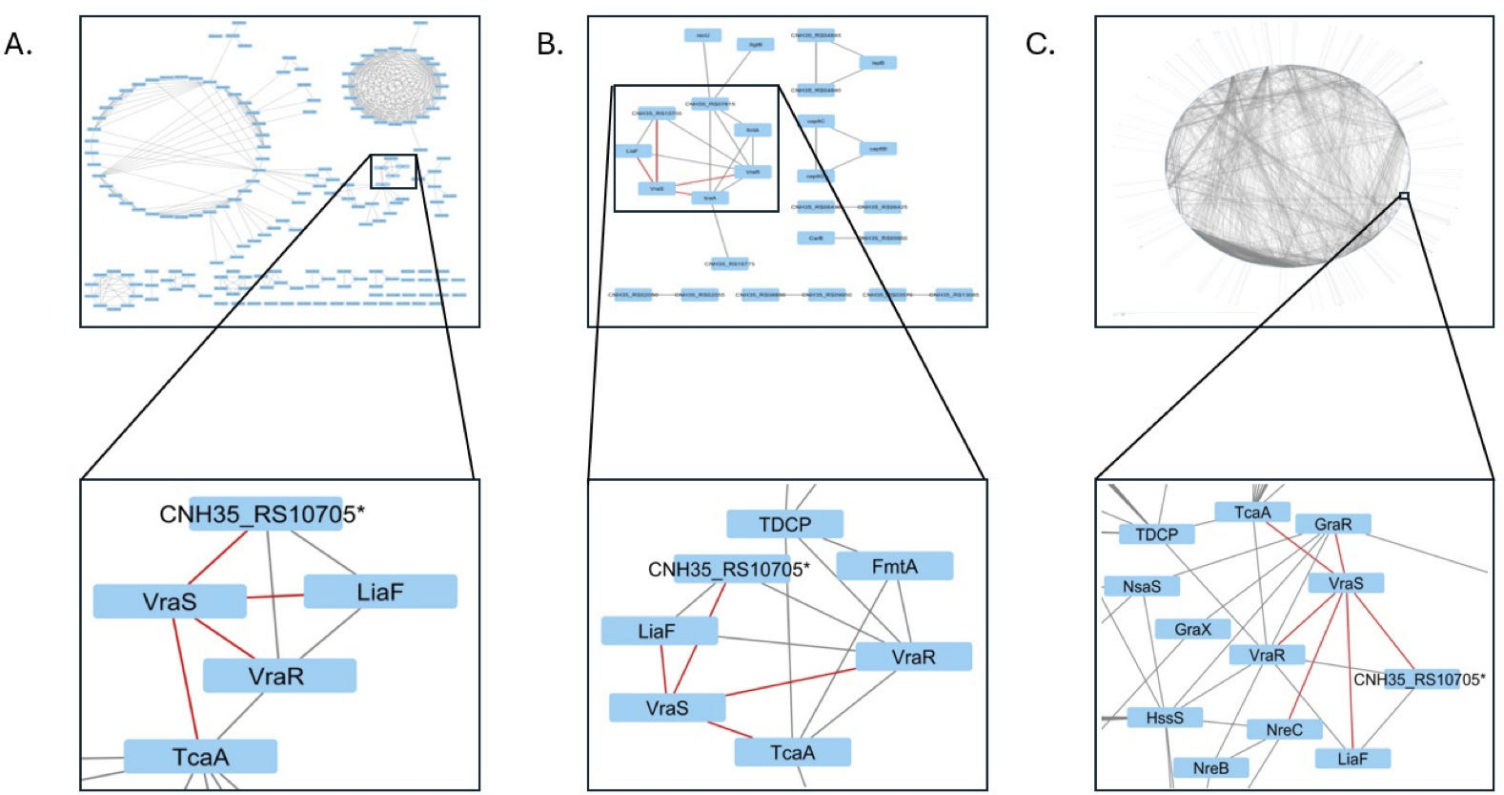
The predicted change in the protein-protein interaction network in *S. aureus* after mutation and/or vancomycin stress as visualized using Cytoscape 3.10.1. The inset shows a zoomed view of the interactions of the VraSR system, and the red lines highlight VraS PPIs. (**A**) PPIs for WT strain after exposure to vancomycin, (**B**) PPIs for T331I compared to WT, and (C) PPIs of T331I after vancomycin compared to T331I.

The STRING analysis can predict the protein–protein interactions (PPIs) that occur in a strain compared to its control, which translates the alterations in gene expression to the proteomics level. The STRING database (http://string-db.org/) was used for the analysis of predicted PPIs to understand the effect of the T33I mutation with and without vancomycin exposure and visualized using Cytoscape 3.10.1 software (30). As a result of the antibiotic stress on the WT strain, a total of 406 PPIs were observed, where VraS showed interactions with VraR, LiaF (VraT), TcaA, and a hypothetical protein (CNH35_RS10705), these constitute VraS “core interactions” (Fig. 6A). In the absence of antibiotics, the T331I mutant showed an additional 28 PPIs compared to the WT strain. VraS in the T331I mutant was able to retain its core interactions, while VraR showed two additional interactions with FmtA (teichoic acid D-Ala esterase) and TDCP (transglycosylase domain-containing protein) (Fig. 6B). When the T331I mutant was exposed to vancomycin, the PPI network became more complex with an additional 4,273 PPIs, a 10-fold increase compared to WT under the same antibiotic stress. Along with its core interactions, VraS showed two additional PPIs with GraR and NreC, while VraR showed three additional interactions with GraR, HssS, and NreB, components in other *S. aureus* TCS signaling network (Fig. 6C). The results suggest that the mutation may have caused a signal crosstalk between VraSR and other two-component systems only under vancomycin stress to facilitate bacterial survival.

## DISCUSSION

In *S. aureus*, VraSR is activated when the bacteria are exposed to external antimicrobials (15). Previous studies have shown that point mutations in *vraS* tend to show up in strains with more resistance against cell wall-inhibiting antibiotics such as vancomycin, teicoplanin, and oxacillin (10, 20, 31–35). Our results show that the T33I mutation affects VraS catalysis, as a 12-fold increase in autophosphorylation rates compared to the WT enzyme was reported, with a threefold increase in the overall catalytic efficiency. The T331I mutation is in close proximity to the ATP-binding site located in the VraS catalytic domain. Mutations in the ATP-binding region can induce significant changes in enzymatic activity and ATP consumption, resulting in increased response regulator phosphorylation levels and ultimately upregulating the cell wall synthesis gene cluster and other regulatory genes (36, 37). The kinase assay used to assess the kinetic rates cycles the produced ADP, thus replenishing ATP in the reaction mixture and preventing VraS dephosphorylation from occurring. This way, the kinetic values obtained are reflective of the autophosphorylation reaction only.

The growth kinetic curves support the presence of a mutant selection window where the mutated strain’s subpopulation (drug-resistant) has the potential to dominate the infection site and enhance bacterial persistence in the presence of drugs. Notably, both the WT and mutant strains exhibited a biphasic growth curve under methicillin treatment. With the WT, the decreased initial growth followed by the surge after 16 h could be attributed to the lower stability of methicillin in aqueous media (38). Yet, the T331I mutant follows the same pattern, with the surge occurring much earlier after 8 h. The growth profile can thus be explained by the increased expression of the *blaZ* lactamases (supported by the qRT-PCR results) much earlier in the mutant strain that hydrolyzes the antibiotic and restores the bacterial growth rates.

The genes encoding resistance mechanisms vary depending on how they are acquired, either on mobile genetic elements or encoded in the bacterial chromosome. *S. aureus* exhibits two prominent resistance mechanisms: beta-lactamase production and PBP2 production (39). Mutations in the VraS lead to an increased expression of the *vraSR* regulon and other cell wall synthesis and resistance-related genes (40). The gene expression levels of *pbp2* and *vraR* were significantly increased in both WT and T331I after vancomycin exposure; however, the effect was not observed in WT with daptomycin or methicillin. We previously reported an increase in expression with vancomycin and no change after carbenicillin treatment for the same Newman strain (24), contrary to what is reported of an increase after oxacillin (41). It seems like the increase in *vraR* expression might be antibiotic dependent rather than a class effect with vancomycin and oxacillin, but not carbenicillin, methicillin, or daptomycin. The T331I strain showed a robust response to daptomycin for both *vraR* and *pbp2* increased expression, which may explain the resistance to daptomycin in the clinical isolate harboring the T331I mutation. Concurrently, the significant changes in *blaZ* expression for T331I explain its resistance to beta-lactam antibiotics.

The differential gene expression of the T331I mutant compared to the WT strain was spread over several pathways, including ribosome and peptidoglycan biosynthesis, and was activated in T331I relative to that in WT. Our results, in agreement with previous findings, indicate that the *vraS* mutation plays an important role in turning on different biological pathways related not strictly to cell wall upregulation but also to bacterial metabolism that significantly impacts antimicrobial susceptibility (8, 42). Therefore, further studies are warranted to determine the role of VraS point mutations in the metabolic pathways we identified to better understand the impact of VraSR on *S. aureus* physiology.

TreP facilitates the conversion of trehalose into trehalose-6-phosphate and correlates with transient drug tolerance and the induction of resistance (43). IsaA, on the other hand, is involved in peptidoglycan hydrolysis (44), and its deletion led to a significant decrease in β-lactam resistance and biofilm formation (45). The upregulation of *treP* and *isaA* in the T331I mutant suggests their possible involvement in the resistant phenotype of the mutant. The genes associated with the histidine biosynthesis pathway (including *hisD* and *hisB*) as well as *cwrA* were significantly downregulated in T331I. Amino acids, such as histidine, arginine, leucine, and valine, are important for the survival of *S. aureus* (46), and the decrease in histidine biosynthesis may correlate with altered metabolism in the mutant. On the other hand, *cwrA* is a VraR-regulated gene encoding a cell wall inhibition-responsive protein, and its decrease was associated with a reduced ability to form a biofilm, resulting in attenuated virulence in S. aureus (47).

The PPI network shows predicted interactions between VraS, VraR, and VraT (a well-known accessory protein for the VraSR system) (48). The *tcaA* and *fmtA* genes are under the transcription control of VraR, and the results imply that an interaction may occur between VraSR and these genes in WT under vancomycin stress and in the T331I mutant regardless of stress. The predicted interaction with TDCP in T331I can result in an increase in the overall transglycosylase activity, which may explain the mutant’s decreased level of *pbp2* expression as one of the major transglycosylase enzymes in *S. aureus* (49). The vancomycin stress on T331I caused additional interactions to appear for VraSR with the other two-component systems, GraSR, HssSR, and NreBC, which implies enhanced co-expression and a concerted response to enhance the mutant’s persistence through possible crosstalk that may occur in the signaling network (35). The computational predictions for PPI do not explicitly account for the subcellular localization of proteins, a critical aspect for assessing the biological relevance of the predicted interactions. While our predictions suggest potential interactions, further experimental validation would be necessary to confirm these interactions.

In conclusion, the T331I mutation in the VraS ATP-binding domain enhances resistance against glycopeptides and beta-lactam antibiotics through several underlying mechanisms, such as increased VraS catalytic ability and large-scale alterations in gene expression across multiple bacterial pathways, including resistance-related genes. Targeting the VraS ATP-binding domain could be an effective strategy against drug-resistant *S. aureus* infections. Since mutations in the VraSR system led to antibiotic resistance, there is a need to develop specific inhibitors that target these mutant forms to overcome the problem of drug resistance in *S. aureus*.

## MATERIALS AND METHODS

### Strains, media, and reagents

All chemicals were obtained from Fisher Scientific unless otherwise stated. Tris-Glycine-SDS was from Invitrogen, Mini-PROTEAN TGX stain-free gel and unstained protein standard ladder were from Biorad. *NdeI and XhoI* restriction enzymes, Instant Sticky-end Ligase Master Mix, and Q5 High-Fidelity DNA Polymerase were from NEB. The drug-susceptible strain *S. aureus* Newman D2C strain (ATCC #25904) and its isogenic mutant VraS T331I were maintained in Luria-Bertani (LB) media at 37°C unless otherwise mentioned. The *Escherichia coli* strain BL21 (DE3) pLysS (Invitrogen #C606010) was used for bacterial heterologous expression of the constructs (all strains used are listed in Table S3).

### Plasmids for protein expression

To generate the His-tagged VraS construct, the previously described VraS-KD fragment was amplified from the pGEX-VraS plasmid (24). Primers 1 and 2 used were designed for amplification to add an *NdeI* restriction site upstream of the VraS-KD gene. The resultant fragment and the pET15b bacterial expression plasmid were treated with *NdeI/XhoI* restriction nucleases for 1 h at 37°C and then ligated using the Ligase Master Mix. The T331I mutation was introduced by site-directed mutagenesis with Q5 high-fidelity polymerase through the generation of two amplified fragments with overlap at the T331I codon switch (primers 3 and 4) following the manufacturer’s protocol. The parent template was removed with DpnI (methylation-dependent endonuclease) treatment, and DH5α cells were transformed with the generated plasmids (pET15b-VraS and pET15b-VraS T331I). Plasmids were then isolated from the positive transformants, and the desired modifications were confirmed by sequencing (primers and oligos are listed in Table S2, and plasmids are listed in Table S4).

### Large-scale heterologous bacterial expression and purification

The pET15b-VraS and pET15b-VraS T331I plasmids were transformed into BL21 (DE3) pLysS cells and incubated overnight at 37°C on LB plates supplemented with ampicillin (100 µg/mL). Positive transformants were cultured in 50 mL of terrific broth supplemented with ampicillin (100 µg/mL) overnight at 37°C and then subcultured to 1 L the next day with shaking at 200 rpm. The cultures were induced at the mid-log growth phase (OD 0.6) with 1 mM IPTG and then incubated overnight at 25°C to allow protein expression before being harvested by centrifugation at 4,000 *g* for 15 min at 4°C. Collected pellets were treated and purified using Ni-NTA affinity chromatography as previously described (25). The T331I mutant was further purified using size exclusion chromatography on a HiLoad 16/600 Superdex 200 (Cytiva) column using a buffer of 50 mM Tris HCl, pH 8, 150 mM NaCl, and 3 mM TCEP. Fractions containing the target protein were pooled and concentrated using Amicon Ultra Centrifugal Filters. The concentration of the protein was determined by 660 nm protein assay (Pierce) using bovine serum albumin as the reference standard.

### Assessment of autophosphorylation rate and Michaelis-Menten kinetics

The reaction rates of VraS and the T331I mutant were tested using a kinetic coupled assay that measures the rate of ATP consumption per published protocols (25). Briefly, the kinase reaction and conversion of ATP to ADP are coupled to a pyruvate kinase/lactate dehydrogenase system that converts NADH to NAD^+^, where the signal monitored was the decrease in NADH absorbance at 340 nm. The reaction rate was monitored over 15 min, and the early linear slope of the reaction (theoretical 10% substrate consumption) was measured. The slopes were mathematically transformed to NADH concentration using the NADH extinction coefficient (6,220 L mol^−1^ cm^−1^). The Michaelis-Menten kinetic constants for the enzymes were assessed using variable concentrations of ATP as previously described (24).

### Construction of T331I *S. aureus* mutant strain

The C992T point mutation in the *vraS* gene was introduced by recombineering coupled with CRISPR-Cas9 counterselection techniques using published protocols (26, 50). Briefly, a spacer sequence for sgRNA, including the *vraS* 992 position, was selected near a specific protospacer adjacent motif (5′-NGG-3′) site. The annealed 20 bp spacer sgRNA with *BsaI* sticky ends (oligos 5 and 6) was inserted into the pCAS9counter vector, followed by transformation into DH5α. The successful insertion was confirmed by PCR using primers 7 and 8 (Table S3) followed by Sanger sequencing. To bypass the *S. aureus* restriction barrier, the resulting plasmid pCAS9counter-T331I was further passed through *E. coli* IM08B strain. An ssDNA of 90 bp recombineering oligo 9 with four phosphorothioate bonds at the 5′ end and T331I alteration, along with four more wobble positions, was designed. The wobble position changes are silent mutations in the protein-coding sequence to avoid the bacterial mismatch repair system. The *S. aureus* Newman D2C strain was turned electrocompetent per published protocols and electroporated with the temperature-sensitive recombineering vector pCN-EF2132tet. The resulting strain, Newman pTet, was turned electrocompetent and used to concurrently electroporate the counterselection vector pCAS9counter-T331I and ssDNA of 90 bp oligonucleotide. Positive transformants were selected from LB agar plates containing chloramphenicol and erythromycin (10 µg/mL each). Screening for mutation was conducted by amplifying the *vraS* gene using primers 10 and 11, and the mutation was confirmed by Sanger sequencing of the amplicon. After sequence confirmation, an isogenic mutant was obtained by streaking on an LB plate (without antibiotics) at 43°C overnight to cure the temperature-sensitive plasmids. The primer sequences are in Table S2, bacterial strains in Table S3, and plasmids in Table S4.

### Drug susceptibility testing

We used the broth microdilution method to determine the MIC of vancomycin, methicillin, and daptomycin (supplemented with 50 mg/L CaCl_2_) against the WT and mutant strains using two different growth media, CAMHB and LB, based on guidelines by the European Committee on Antimicrobial Susceptibility Testing as previously described (24). Briefly, the strains were grown to the logarithmic phase in the media, turbidity was adjusted to 0.5 McFarland standard, followed by 1,000-fold serial dilution to prepare the inoculum with the bacterial density of ~10^5^ CFU/mL. The cultures were co-incubated with the antibiotics at a final drug concentration ranging between 0.25 and 32 mg/L. At the end of 24 h of incubation at 37°C, plates were visually inspected, and the drug concentrations completely inhibiting the bacterial growth were recorded as MIC.

### Concentration-response and growth kinetics studies

We performed a dose-response study with both strains in CAMHB, in a total volume of 5 mL. The drug concentrations and the inoculum preparation were the same as described above. After 24 h of co-incubation with the drugs at 37°C under shaking conditions, the cultures were washed twice with normal saline to remove the carry-over drug, serially diluted, and spread on Muller-Hinton agar. Cultures were incubated for 24 h before the CFU/mL with each drug concentration was recorded. GraphPad Prism (version 10) was used for data analysis. Growth rates of the WT and T331I mutant strains were determined in LB. Cells were incubated at 37°C with constant shaking in a microplate reader (Synergy H1, BioTek, USA), and the OD_600_ was measured for 24 h at 30 min intervals as previously described (24). All experiments were performed twice in replicates of three.

### Quantitative real-time RT-PCR

The strains were cultured to an OD_600_ of 0.6 and then either LB or selected antibiotics at 2× MIC level were added for 1 h before harvesting. Total RNA extraction was carried out, and the extracted RNA was used for cDNA synthesis to conduct qRT-PCR for target genes according to published protocols (24). The data were normalized to the threshold cycle (Ct) value of housekeeping gene 50S ribosomal protein L4 (*rplD*), and the genes’ expression level was obtained using the 2^−∆∆*C*t^ method (primer sequences are listed in Table S2). Statistical analyses were performed using Prism (version 10) using two-way ANOVA with *post hoc* multiple comparisons to determine the statistical significance.

### RNA-seq analysis

The total extracted RNA from four samples (i) New_Van (WT with vancomycin), (ii) T331I_Van (mutant with vancomycin), (iii) New (WT control), and (iv) T331I (control) was sent to Novogene (Sacramento, CA, USA) for DGE analysis. The fragments per kilobase of exon model per million mapped fragments of each gene were calculated using the Feature Counts version 1.5.0-p3 for quantitative analysis by mapping to Newman’s genome as a reference. The DGE analysis was performed using Edge R software, and statistical tests were performed to identify genes with significant up- or downregulation compared to the Newman WT strain. The resulting *P*-values were adjusted using Benjamini and Hochberg’s approach to control the false discovery rate. The *P*-value < 0.05 and |log2 (fold change)| value > 1 were set as the threshold for significantly differential expression. For qRT-PCR validation of the top dysregulated genes, the primers were designed using the PrimerQuest tool from Qiagen and validated to ensure specificity by ensuring single peaks are obtained in the melt curve and confirmatory PCR and DNA gel resulting in a single product per reaction (Fig. S5A). Different primer concentrations (1, 10, 30, and 100 µM) were screened to ensure the sensitivity; the qRT-PCR cycle threshold (Ct) was the lowest in the 10–100 µM range. To ensure linear detection, calibration curves were built by screening different concentrations of template cDNA (0.5, 1, 10, and 100 ng/µL) with 10 µM primers. The coefficient of determination (*R*2) for tested primers was >0.92, indicating good reliability (Fig. S5B).

## Data Availability

The RNA-seq data generated in this study are publicly accessible through the NCBI Sequence Read Archive (SRA) under accession number PRJNA1227456.

## SUPPLEMENTAL MATERIAL

Data S1 spectrum.00095-25-s0001.xlsx List of dysregulated genes in the 4 compared samples.

Data S2 spectrum.00095-25-s0002.xlsx List of genes in the 15 comparisons shown in Figure S2.

Supplementary Material spectrum.00095-25-s0003.docx Fig. S1 to S5; Table S1 to S4.

ASM does not own the copyrights to Supplemental Material that may be linked to, or accessed through, an article. The authors have granted ASM a non-exclusive, world-wide license to publish the Supplemental Material files. Please contact the corresponding author directly for reuse.
