# Supplementary material for: A mechanistic understanding of the effect of Staphylococcus aureus VraS histidine kinase single-point mutation on antibiotic resistance
Source: Microbiol Spectr. 2025 Apr 15;13(5):e00095-25. doi: 10.1128/spectrum.00095-25 (PMC12054033; doi:10.1128/spectrum.00095-25)
Supplement: Supplementary Material — Fig. S1 to S5; Table S1 to S4. [file spectrum.00095-25-s0003.docx]

**Supplementary Information**

**Figure S1.** **Purification of VraS WT and T331I mutant.** Raw SDS-PAGE images of A) VraS WT fractions and B) T331I mutant fractions from the His-Trap column, and C) T331I mutant fractions from the gel filtration column (M: molecular weight standard ladder, L: load pre-column, F: flowthrough, W: wash, Elution represent fraction in the protein eluted peak, Z1-3: representative fractions from the gel filtration column).

**A)**


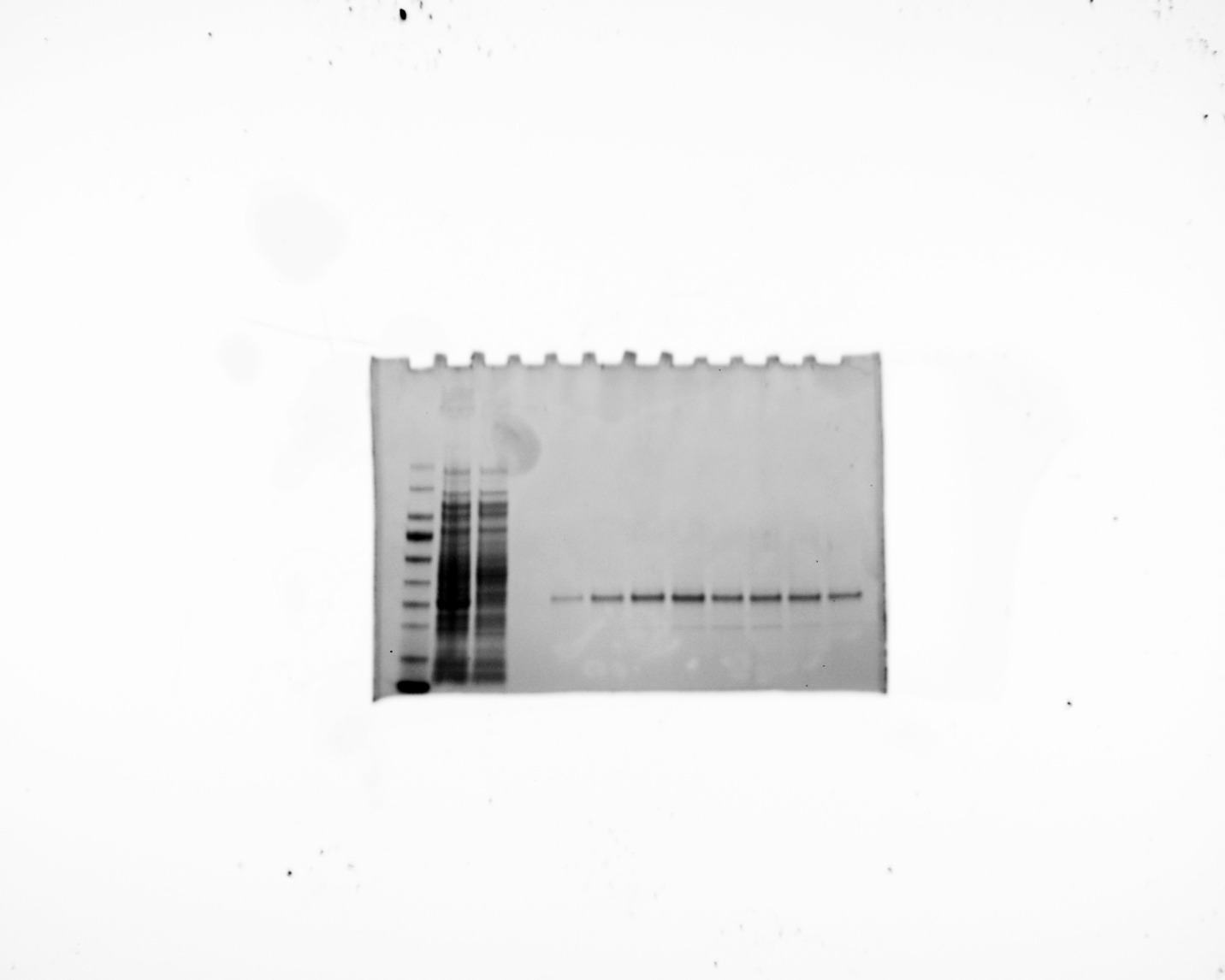


| M | L | F | W | Elution |
| --- | --- | --- | --- | --- |

43

34

**C)**

**B)**

| M | Z1 | Z2 | Z3 | x | L |
| --- | --- | --- | --- | --- | --- |


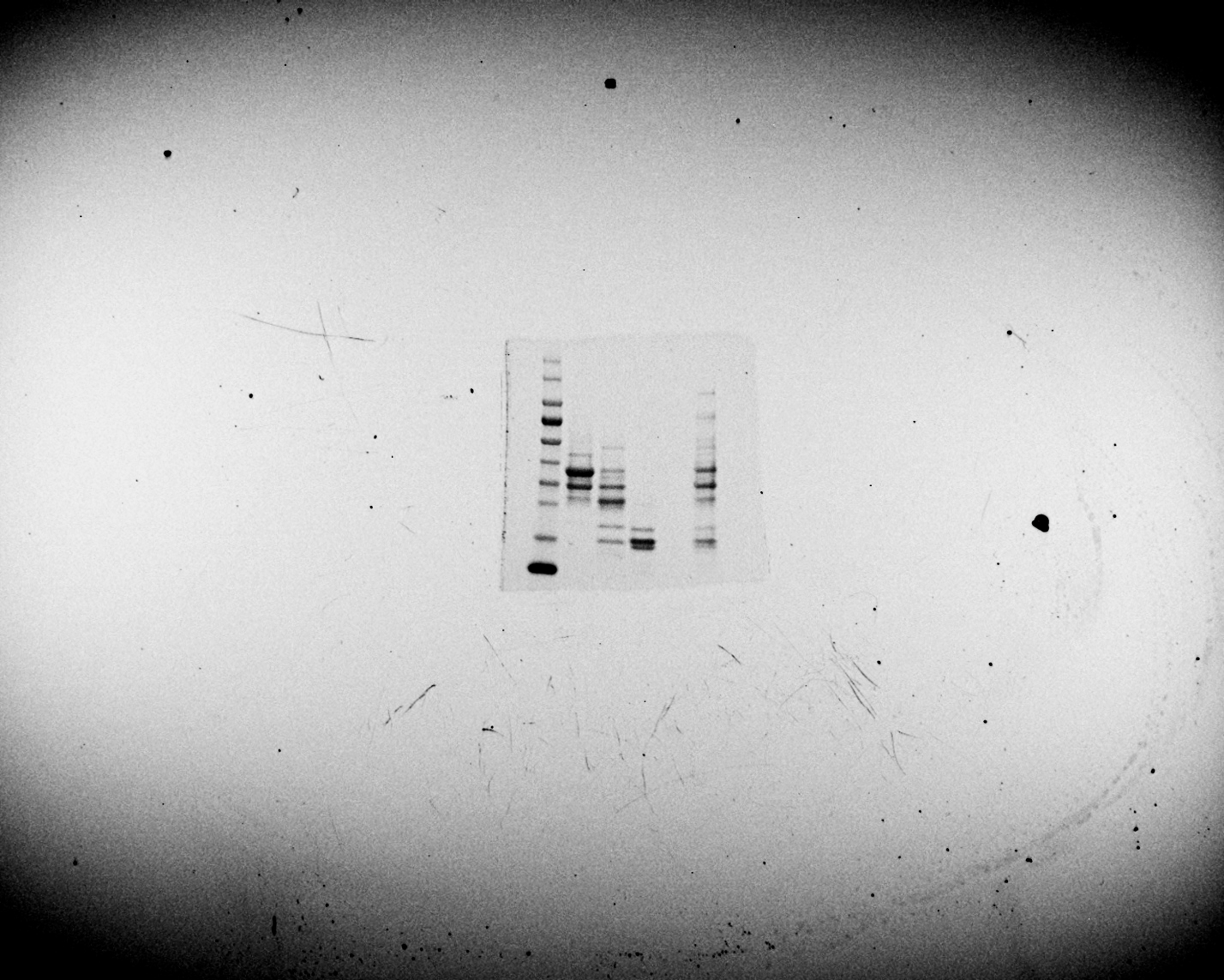


| M | M | L | F | W | Elution |
| --- | --- | --- | --- | --- | --- |

43

34

43

34

**Table S1. Inhibitory sigmoid model parameters comparing drug’s efficacy and potency between WT and T331I mutant strain.** Data is presented as mean + SE (standard error).

|  | Vancomycin | | Methicillin | | Daptomycin | |
| --- | --- | --- | --- | --- | --- | --- |
|  | WT | T331I | WT | T331I | WT | T331I |
| Best-fit values | | | | | | |
| Econ | 6.12 ± 0.17 | 6.61 ± 0.10 | 5.65 ± 0.13 | 5.99 ± 0.09 | 6.18 ± 0.12 | 5.57 ± 0.20 |
| Emax | 6.13 ± 0.29 | 5.63 ± 0.27 | 4.28 ± 0.39 | 3.07 ± 0.30 | 6.36 ± 0.21 | 6.41 ± 0.69 |
| H | 30.16 ± 45.11 | 31.65 ± 10.26 | 2.52 ± 0.71 | 4.31 ± 1.17 | 2.38 ± 0.27 | 1.19 ± 0.22 |
| EC | 1.28 ± 0.05 | 1.66 ± 0.03 | 7.44 ± 1.03 | 11.14 ± 1.21 | 3.10 ± 0.17 | 5.10 ± 1.03 |
| Goodness of Fit | | | | | | |
| R squared | 0.98 | 0.99 | 0.93 | 0.91 | 0.99 | 0.96 |


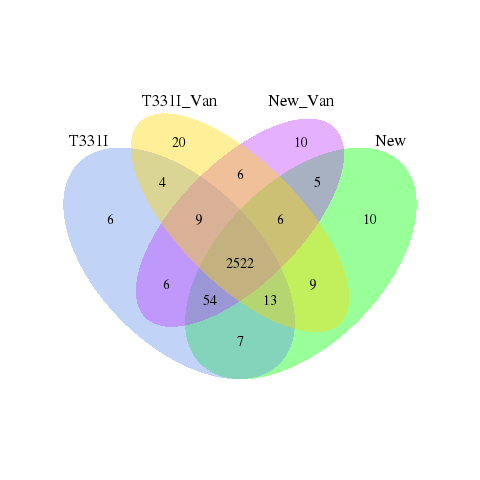
**Figure S2.** Genes expression data showing 15 comparisons of similarity between the transcription profile of T331I mutant compared to WT strain (New) with and without vancomycin (Van) stress. For example, 20 genes were found to be uniquely expressed in the T331I strain only after vancomycin exposure (the list of genes is found in supplementary material).

**Table S2.** Primers and oligonucleotides used in this study.

| **Number** | **Primers/**  **Oligonucleotides** | **Sequence (5′-3′)^#^** | **Reference** |
| --- | --- | --- | --- |
| 1 | VraS add Nde1-F | CAGCGGCCATATGGAGGATCTGTACTTTCAGAGCTGCATGGAGGGTGAAACCG | This study |
| 2 | VraS add Nde1-R | CAGTCAGTCACGATGCGGCC | This study |
| 3 | T331I-F | GCCGGACAGCGGTATCCGTATTG | This study |
| 4 | T331I-R | CCTTAACCTCAATGGATACCGCTGTC | This study |
| 5 | VraS-gRNA-F | **AGCTC** AGATTCAGGTACACGTATCG G | This study |
| 6 | VraS-gRNA-R | **AAAAC** CGATACGTGTACCTGAATCT G | This study |
| 7 | sgRNA_check_F | AAAAATATGAACACTCTATCATTG | 1 |
| 8 | sgRNA_check_R | CGGTGCCACTTTTTCAAGTT | 1 |
| 9 | T331I 90 bps oligo^$^ | t*g*g*a*aattggtgcaacgttccatattgtatcattgccagattcTggAaTacgAatTgaggtgaaagcacctttaaataaggaggattcgt | This study |
| 10 | vraS-F | AGTGCCAATGAAAGTTGTGC | This study |
| 11 | vraS-R | CTGGCTTCAACTCATGGGC | This study |
| 12 | pbpB-F | CAAGCAACAGATCCTCACCCT | 2 |
| 13 | pbpB-R | AATCGCATGGTTTGTTGCCC | 2 |
| 14 | rplD-F | TTCGGACCAACTCCAAGA | 3 |
| 15 | rplD-R | CGAGCACCTCCTCAAC | 3 |
| 16 | vraR-F | GCGCGCTTTTTCATACGGTT | 4 |
| 17 | vraR-R | ATCGCCGATGCAGTTCGTAA | 4 |
| 18 | blaZ-F | GCTTTAAAAGAACTTATTGAGGCTTCA | 5 |
| 19 | blaZ-R | CCACCGATYTCKTTTATAATTT | 5 |
| 20 | treP-F | CAG CTT AGG CGG TAC GTA TTT | This study |
| 21 | treP-R | GAT GTT AAT GCC AAG CCT TCT TC | This study |
| 22 | isaA-F | TTC TAC ATG GGC TGC AAT CA | This study |
| 23 | isaA-R | CAA CCT GGC ATA GTT TGG AAT AAA | This study |
| 24 | cwrA-F | ACA GGC ACA GTT GCT ATC TT | This study |
| 25 | cwrA-R | GAC TCG TGT CGT TTG TCA TTT C | This study |
| 26 | hisD-F | TGA CAC CAC CTC AAC CTA ATG | This study |
| 27 | hisD-R | GAG CAC CAC CAA CTT GAA ATA C | This study |
| ^#^Bold font represent the *BsaI* cutting site  ^$^Capital letters represent the altered bases for CRISPR counterselection, the base in red represent the mutated base, the stars indicate modified phosphorothioate bonds. | | | |

**Figure S3.** Fold change in expression levels of selected top dysregulated genes identified from the RNA-seq data. The data represent the mean ± SE (*n* = 3), and statistical significance was calculated via one-way ANOVA with multiple comparisons using GraphPad PRISM.

**Figure S4.** KEGG pathway analysis of DEGs comparing T331I mutant and Newman wild type, both under vancomycin stress. The dot size indicates the number of DEGs enriched in the pathway. The color scale indicates the statistical significance of the change in the enrichment level (padj).

**Table S3**. Strains used in this study.

| **Strain** | **Description** | **Source** |
| --- | --- | --- |
| ***S. aureus*** |  |  |
| Newman D2C | WT Reference strain | ATCC #25904 |
| Newman pTet | Newman strain carrying recombineering plasmid | This study |
| T331I | Newman strain carrying T331I mutation | This study |
|  |  |  |
| ***E. coli*** |  |  |
| DH5α | Routine cloning host for DNA preparations | Thermo #18-258-012 |
| IM08B | *S. aureus* methylation machinery, SA08BWPN25-hsdS (CC8-1) (SAUSA300_0406) of NRS384 integrated between the essQ and cspB | BEI Resources #NR-49806 |
| BL21 (DE3) pLysS | Bacterial heterologous expression of constructs *F^–^ ompT hsdSB (rB^–^, mB^–^) gal dcm (DE3) pLysS (Cam^R^)* | Thermo #C606010 |

**Table S4**. Plasmids used in this study

| **Plasmid** | **Description** | **Reference** |
| --- | --- | --- |
| pET15b-VraS | VraS with N-terminally 6xHis-tagged protein, thrombin site, Amp^r^, lacI promoter. | This study |
| pET15b-VraS T331I | VraS-T331I with N-terminally 6xHis-tagged protein, thrombin site, Amp^r^, lacI promoter. | This study |
| pCas9counter | Ery^r^, expresses modifiable sgRNA and cas9, temperature sensitive | 1 |
| pCas9counter-T331I | Ery^r^, sgRNA designed to target PAM near T331I in VraS, temperature sensitive | This study |
| pCN-EF2132tet | Chl^r^, expresses recombinase EF2132 for incorporating ssDNA oligonucleotides into Newman genome, temperature-sensitive | 1 |

**Figure S5: RT-PCR Primer Validation**. A) DNA gel picture showing single bands after PCR amplification of 5 ng cDNA of WT strain using 10 µM of the primers. Lane 1 is the 100bp DNA ladder, lane 2 is *treP*, lane 3 is *isaA*, lane 4 is *cwrA*, and lane 5 is *hisD* PCR reaction products. B) calibration curves showing the correlation between log cDNA concentration of the samples (10 – 100 ng/µL) and the qRT-PCR cycle threshold (Ct) using 10 µM of the primers. The experiment was done in duplicate for each concentration, and the graphs show the Coefficient of Determination (r^2^) of each standard curve.

| 1 | 2 | 3 | 4 | 5 |
| --- | --- | --- | --- | --- |

**
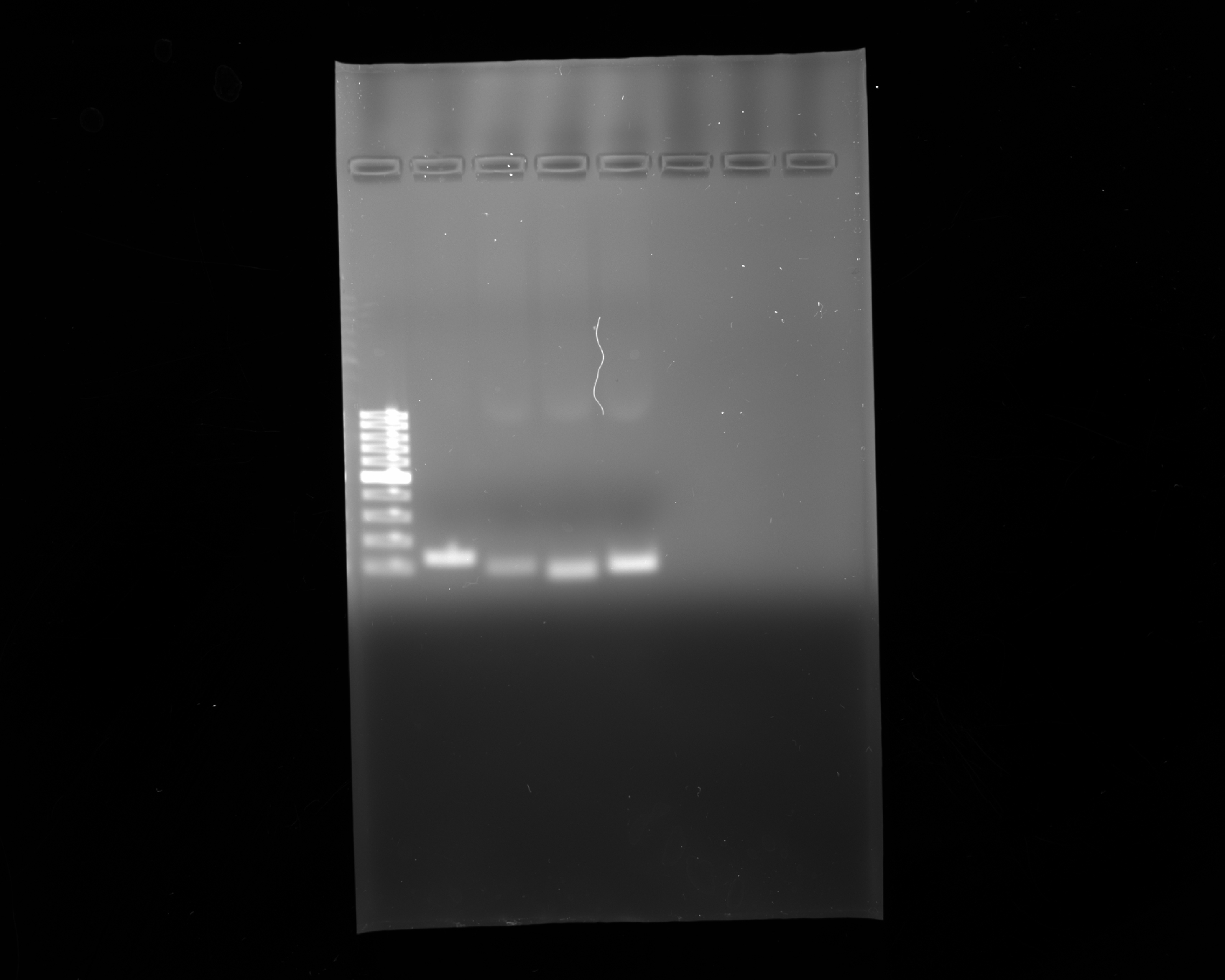
**

**A**

**B**

**References**

1. Penewit K, Holmes EA, McLean K, Ren M, Waalkes A, Salipante SJ. 2018. Efficient and Scalable Precision Genome Editing in *Staphylococcus aureus* through Conditional Recombineering and CRISPR/Cas9-Mediated Counterselection. mBio 9:10.1128/mbio.00067-18.
2. Gillard, K., Miller, H. B., & Blackledge, M. S. (2018). Tricyclic amine antidepressants suppress β‐lactam resistance in methicillin‐resistant Staphylococcus aureus (MRSA) by repressing mRNA levels of key resistance genes. *Chemical Biology & Drug Design*, *92*(5), 1822-1829.
3. Sihto, H. M., Tasara, T., Stephan, R., & Johler, S. (2014). Validation of reference genes for normalization of qPCR mRNA expression levels in Staphylococcus aureus exposed to osmotic and lactic acid stress conditions encountered during food production and preservation. *FEMS Microbiol. Lett.*, 356(1), 134-140.
4. Cutrona, N., Gillard, K., Ulrich, R., Seemann, M., Miller, H. B., & Blackledge, M. S. (2019). From antihistamine to anti-infective: loratadine inhibition of regulatory PASTA kinases in Staphylococci reduces biofilm formation and potentiates β-lactam antibiotics and vancomycin in resistant strains of Staphylococcus aureus. *ACS Infect. Dis*., 5(8), 1397-1410.
5. Pereira, L. A., Harnett, G. B., Hodge, M. M., Cattell, J. A., and Speers, D. J. (2014) Real-time PCR assay for detection of blaZ genes in Staphylococcus aureus clinical isolates, *J Clin Microbiol* 52, 1259-1261.
